# A Novel Educational Control Group Mobile App for Meditation Interventions: Single-Group Feasibility Trial

**DOI:** 10.2196/19364

**Published:** 2020-07-21

**Authors:** Jennifer Huberty, Ryan Eckert, Megan Puzia, Breanne Laird, Linda Larkey, Ruben Mesa

**Affiliations:** 1 College of Health Solutions Arizona State University Phoenix, AZ United States; 2 Mays Cancer Center University of Texas Health San Antonio MD Anderson San Antonio, TX United States; 3 Behavioral Research and Analytics, LLC Salt Lake City, UT United States; 4 Edson College of Nursing and Health Innovation Arizona State University Phoenix, AZ United States

**Keywords:** feasibility, smartphone, mHealth, digital health, cancer, beta test

## Abstract

**Background:**

Smartphone ownership is becoming ubiquitous among US adults, making the delivery of health interventions via a mobile app (ie, mobile health [mHealth]) attractive to many researchers and clinicians. Meditation interventions have become popular and have been delivered to study participants via mobile apps to improve a range of health outcomes in both healthy adults and those with chronic diseases. However, these meditation mHealth interventions have been limited by a lack of high-quality control groups. More specifically, these studies have lacked consistency in their use of active, time-matched, and attention-matched control groups.

**Objective:**

The purpose of this study is to beta test a novel health education podcast control condition delivered via a smartphone app that would be a strong comparator to be used in future studies of app-based meditation interventions.

**Methods:**

Patients with myeloproliferative neoplasm (MPN) cancer were recruited nationally. Upon enrollment, participants were informed to download the investigator-developed health education podcast app onto their mobile phone and listen to ~60 min/week of cancer-related educational podcasts for 12 weeks. The benchmarks for feasibility included ≥70% of participants completing ≥70% of the prescribed 60 min/week of podcasts, ≥70% of participants reporting that they were satisfied with the intervention, and ≥70% of participants reporting that they enjoyed the health education podcasts.

**Results:**

A total of 96 patients with MPN were enrolled in the study; however, 19 never began the intervention. Of the 77 patients who participated in the intervention, 39 completed the entire study (ie, sustained participation through the follow-up period). Participation averaged 103.2 (SD 29.5) min/week. For 83.3% (10/12) of the weeks, at least 70% of participants completed at least 70% of their total prescribed use. Almost half of participants reported that they enjoyed the health education podcasts (19/39, 48.7%) and were satisfied with the intervention (17/39, 43.6%). There were no significant changes in cancer-related outcomes from baseline to postintervention.

**Conclusions:**

A 12-week, health education podcast mobile app was demanded but not accepted in a sample of patients with cancer. Using the mobile app was not associated with significant changes in cancer-related symptoms. Based on findings from this study, a health education podcast mobile app may be a feasible option as a time- and attention-matched control group for efficacy trials with more extensive formative research for the content of the podcasts and its acceptability by the specific population.

**Trial Registration:**

ClinicalTrials.gov NCT03907774; https://clinicaltrials.gov/ct2/show/NCT03907774

## Introduction

Smartphone ownership is becoming ubiquitous among adults in the United States (81% in 2019) [1]. Using mobile devices to support health and wellness (ie, mobile health [mHealth]) [2] may be a promising approach to help individuals prevent or manage chronic conditions and improve health outcomes [3]. However, many studies of mHealth interventions, particularly mobile app interventions, have been substantially limited by the lack of high-quality comparators (ie, control conditions). The most common types of control conditions include usual care (ie, usual care for the critical condition), wait-list control (ie, usual care and will later receive the intervention), and active control (ie, control group receives an activity or intervention that controls for some aspect of attention, time, or expectation) [4]. Active control groups can be more effective than wait-list control groups [5]. However, there is a lack of research on active control groups in app-based interventions [6,7]. Recent reviews and meta-analyses have called for improvements in the design of control groups within randomized control trials that evaluate the efficacy of mobile app interventions [4,8], and the National Institutes of Health (NIH) has recommended that careful selection of a comparator be designed to reflect the *primary purpose* of the study [9]. It is clear that there is a need for active *time- and attention-matched* comparators. That is, control conditions must aim to not only match the mode of activity or delivery of the intervention but also match the time and attention that is spent on the intervention. Without active control conditions that match interventions with regard to time and attention, studies of mHealth interventions are unavoidably confounded by differences in participant engagement [9]. There is a need to design and explore the feasibility of active time- and attention-matched control groups for mHealth studies.

Meditation apps have become quite popular in recent years [4,10] and have been used to improve mental and physical health in a range of healthy [7,11-15] and health-compromised populations [16,17]. However, few mobile app meditation studies have used active, time-matched, and attention-matched comparators [7,13,14,18] even when the study primarily aimed to determine effectiveness or efficacy. The most common comparators for these studies have been wait-list control groups, usual care, and educational handouts [12,15]. For example, in our work using a meditation app to reduce symptom burden in patients with hematological cancer, we used an educational handout with information about managing fatigue in cancer as our control group. Participation in the control group was not associated with improvements in health or cancer-related symptoms, suggesting that the cancer-related educational content may be reasonable for a control condition; however, this group did not match the engagement level of our intervention participants. This type of comparator could be improved by modifying it to mirror the basic functionality, look, and feel of the intervention group’s meditation app (ie, active); match the time that the intervention group spends participating in meditation (ie, time-matched); and match the attention and basic mode of delivery that the intervention group requires to meditate (ie, attention-matched).

We sought to develop and beta-test an appropriate comparator app for interventions using a mobile meditation app. We developed the app with the ability for content to be modified, added, and updated, and be used across various populations participating in mobile meditation interventions. To further our progressive line of research, we chose to conduct the beta test in patients with hematological cancer (specifically, myeloproliferative neoplasm [MPN]) due to our ongoing work with this population and our partnerships with foundations in which to recruit patients with cancer for our beta test. Therefore, the purpose of this study is to beta test a novel health education podcast control condition delivered via a mobile app that would be a strong comparator to be used in future studies of app-based meditation interventions. We hypothesized that implementing the health education podcasts in a sample of patients with MPN would be feasible (ie, demanded, accepted) and that using the podcasts would not be associated with significant improvements in health, cancer-related symptoms (ie, depression, anxiety, pain intensity, and sleep disturbance), or total symptom burden. Our benchmarks for success were ≥70% of participants completing ≥70% of the prescribed 60 min/week of podcasts, ≥70% satisfied with the intervention, and ≥70% enjoying the health education podcasts.

## Methods

This study was approved by the Institutional Review Board at Arizona State University.

### Recruitment and Enrollment

Guided by Bowen and colleagues’ [19] recommendations for designing feasibility studies, we aimed to enroll 100 participants in the study [19]. Because these early trials are used, in part, to estimate effect size and power for future trials, we did not expect to be fully powered to detect changes in primary study outcomes. Participants were recruited nationally via internet-based strategies, including social media (ie, Facebook, Twitter), social networking sites, and online and email listservs. All recruitment methods were approved by the Arizona State University Institutional Review Board. The study was advertised as a mobile app health education intervention. We recruited self-reported patients with hematological cancer (ie, patients with MPN) because we have a progressive line of work involving patients with MPN using mindfulness approaches to reduce symptom burden (eg, yoga, meditation). However, the app was developed to be applied in app-based meditation interventions across various populations. Interested participants completed an eligibility link on REDCap (Vanderbilt University). Inclusion criteria were as follows: (1) had a diagnosis of MPN (ie, polycythemia vera, myelofibrosis, essential thrombocythemia) identified by a treating physician, (2) had access to a smartphone on a regular basis, (3) had access to reliable home internet, (4) could read and understand English, and (5) were 18 years or older. The exclusion criteria were as follows: (1) planned change in pharmacologic intervention (ie, new drug, bone marrow transplant) during the study interval (ie, 12 weeks) and (2) resided outside of the United States. If eligible, the patients were emailed a link to a video explaining the informed consent and study procedures. If interested, patients were asked to respond to the email indicating that they reviewed the video and had the opportunity to ask questions. Patients then completed an electronic informed consent delivered via REDCap prior to participation. If ineligible, patients were sent an email notification thanking them for their interest in the study and to respond if they were interested in being notified about future studies.

### Research Design

The study was a single-group, *beta test study* of a new control intervention to be used as a comparator in future studies involving mindfulness and meditation mobile apps. A total 96 participants were enrolled in the study and assigned to a health education podcast group.

#### Podcast Control App Development

**Figure 1:**
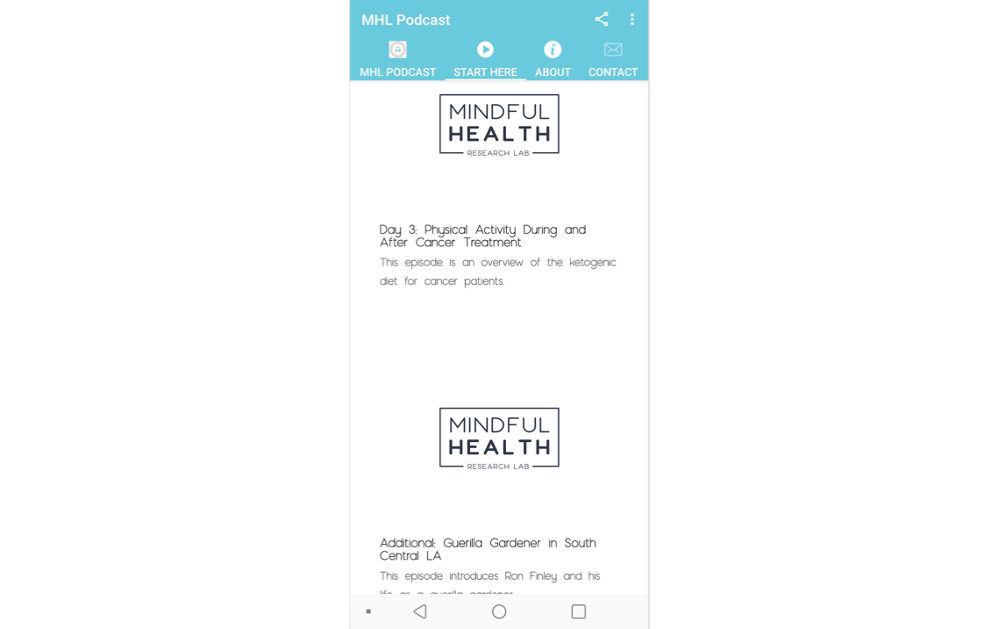
Mindful Health Lab (MHL) podcast app screenshot.

The concept for the podcast control app was developed by a PhD-level mHealth researcher with expertise in the development of national, digitally delivered interventions to improve physical and mental health. The goal was to develop an app that could deliver education information in the same context that a consumer-based mindfulness meditation app delivers content (ie, log onto an app, click on the content, listen). See Figure 1 for a screenshot of the Mindful Health Lab (MHL) podcast control app. The podcast control app was developed to match time (ie, 60-70 min/week) and attention (ie, same context of delivery and same functionality) of some mobile app meditation interventions [6,7,15]. We used the mobile app Calm as our model because of our long-standing partnership and research being conducted with the app [15,16]. The app was also developed so that content could be changed and tailored to any population in future studies. For example, if the study team was conducting a study in college students with the Calm app, the podcast health education content could be modified to be specific to college students. In the case of this study, we used patients with cancer. Thus, content was tailored to health education for patients with cancer. The app was designed to have the same general features as the Calm app (eg, reminders to listen to podcasts, ability to share use on social media, ability to track time spent listening to podcasts) but without the same branding. We did not include similar branding as Calm to keep control participants blinded to the app that would be used in the intervention group.

To gather content for the app, the research team searched publicly available podcasts related to health education through credible government and higher education websites and podcasts that could be used with our target population for our beta test (ie, patients with cancer). The selected content was then uploaded to an app created by a developer. The podcast content was only used for noncommercial purposes with credit to the source of the content included in the app. The audio was not changed or altered in any way and only those enrolled in the study had access to the content. The type of specific educational content included a variety of topic areas including nutrition, physical activity, time, and stress management, as well as general wellness and life-related topics (eg, cultivating happiness, organization practices).

#### Podcast Control App Prescription

Participants were asked to listen to the health education podcasts on their smartphone for approximately 60 min/week for 12 weeks. Podcasts were arranged by week and by day. There were approximately two to three podcasts prescribed per week, averaging about 22 minutes per podcast. Additionally, there were one to three podcasts offered per week for participants to complete if they wanted to listen to more than 60 min/week. Although navigation through the podcast prescription was suggested by week, participants were not restricted from skipping around within the weeks. Time spent listening to the podcasts was collected by the app and downloaded by the research team.

#### Outcomes

The a priori benchmarks for feasibility were based on Bowen and colleagues [20] feasibility criteria and included ≥70% of participants completing ≥70% of the prescribed 60 min/week (42 min/week) of podcasts (ie, demand), ≥70% of participants reporting that they were satisfied with the intervention, and ≥70% of participants reporting that they enjoyed the health education podcasts (ie, acceptability). These specific benchmarks have been used successfully in other recent feasibility studies [16,21].

Questionnaires were administered at baseline (week 0), midintervention (week 6), and postintervention (week 12). These questionnaires included demographics (baseline only), satisfaction-related questions developed by the researchers (week 12 only), NIH Patient-Reported Outcomes Measurement Information System (PROMIS) outcomes (global health, pain intensity, anxiety, depression, and sleep disturbance), and the MPN Symptom Assessment Form Total Symptom Score (MPN-SAF TSS). The satisfaction questionnaire asked questions related to enjoyment, satisfaction, recommendation to others, etc (see Textbox 1 for satisfaction survey questions and responses). Answers were either a yes or no format, or a 5-point Likert scale. The NIH PROMIS is a valid and reliable tool for the measurement of symptoms among patients with cancer [22-25]. The MPN-SAF TSS is a valid and reliable way of assessing total symptom burden among patients with MPN [5]. All participants were provided with a US $25 digital gift card for completion of all questionnaires.

**Postintervention satisfaction survey questions.**

1. On a scale of 1 to 5 (1=did not enjoy at all, 5=very much enjoyed), how would you rate your overall enjoyment of listening to the podcasts?
2. On a scale of 1 to 5 (1=not at all satisfied, 5=very much satisfied), how would you rate your overall satisfaction with the podcasts?
3. Would you recommend that other patients with myeloproliferative neoplasm (MPN) listen to the 12-week podcast prescription?
  Yes
  No
4. Do you feel like you learned something about your MPN, or about cancer in general, that you did not know before starting the study?
  Yes
  No
5. Have you made any changes to your normal daily activities because of something that you learned in the podcast?
  Yes
  No
6. Did you experience any limitations while trying to access the podcasts?
  No, none
  Bad or slow internet connection
  Hard to hear or view the podcasts
  Smartphone broken
  Other, please describe: [free response textbox]
7. Do you have anything else you would like to share with us in regard to your participation in the MPN podcast study?
  [Free response textbox]

### Statistical Analysis

All analyses were conducted using SPSS 26.0 (IBM Corp). Descriptive statistics were used to characterize participants’ app-use patterns over time, and frequency data from the satisfaction survey were used to describe participants’ perceptions of the podcasts and the prescription schedule. Changes in PROMIS outcomes and MPN-SAF TSS were assessed using multivariate analyses of variance.

## Results

### Recruitment and Enrollment

**Figure 2:**
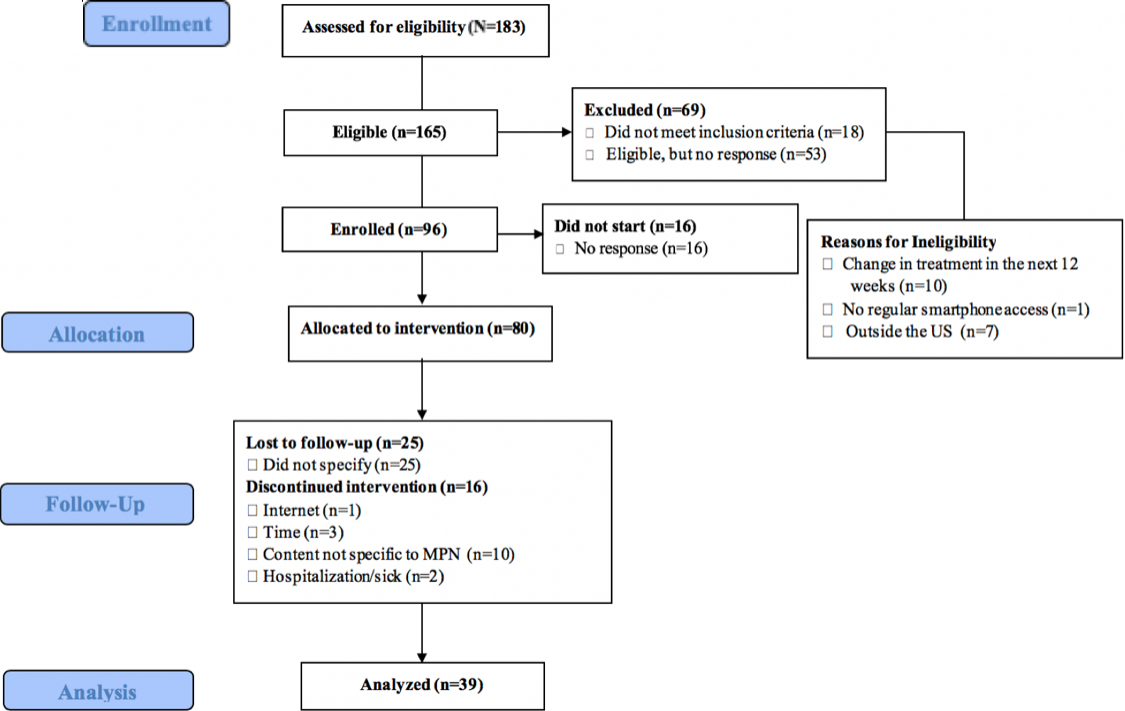
Study consort diagram. MPN: myeloproliferative neoplasm.

Initially, 96 patients with MPN were enrolled in the study; however, 19 (19.8%) never began the intervention without disclosing their reasoning and without responding to contact attempts (see Figure 2). Of the 80 patients who participated in the intervention, 39 (48.8%) completed the entire study (ie, sustained participation through the follow-up period). There were 28 participants lost to follow-up that never responded to the three contact attempts after initial enrollment and 16 that dropped out and provided the research team with a specific reason for discontinuing the study. The reasons for dropout included content not being MPN-specific (n=10), lack of time (n=3), internet connectivity issues (n=1), or illness or hospitalization (n=2).

**Figure 3:**
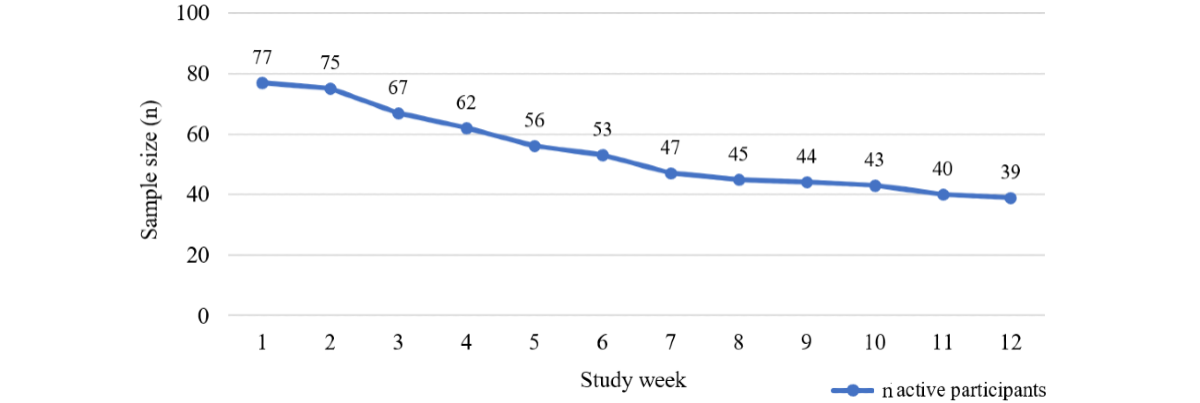
Participation and attrition by week during the intervention period.

Enrollment by week is presented in Figure 3. Analyses included data from all participants who were enrolled in the study through the point that they stopped using the app, defined as the point at which participants did not engage with the app for any subsequent weeks during the intervention period. For example, if a participant used the app during weeks 1, 2, 3, and 8, they were included in adherence analyses for weeks 1-8 (with use during weeks 4-7 calculated as 0 minutes) but were not included in analyses after the point when they stopped using the app (weeks 9-12), at which point they were considered to have discontinued study participation.

As shown in Table 1, the sample was predominately White, non-Hispanic, and female. The average age was 56.1 (SD 10.9) years. Most participants had earned a higher education degree, were married, and had an annual household income more than US $61,000. Study noncompleters were demographically similar to those who completed the study.

**Table 1:** Demographic characteristics of the sample.

	Category		Completed study (n=39), n (%)	Did not complete study (n=38), n (%)
	**Gender**			
		Female	37 (94.9)	33 (86.8)
		Male	2 (5.1)	5 (13.2)
	**Race**			
		White	38 (97.4)	35 (92.1)
		Black/African American	0 (0.0)	1 (2.6)
		Asian	1 (2.6)	1 (2.6)
		American Indian	0 (0.0)	2 (5.3)
		Other	1 (2.6)	1 (2.6)
	**Ethnicity^a^**			
		Non-Hispanic	38 (97.4)	37 (100.0)
		Hispanic	1 (2.6)	0 (0.0)
	**Education**			
		High school/GED^b^	2 (5.1)	2 (5.3)
		Some college	3 (7.7)	10 (26.3)
		Associate’s degree	1 (2.6)	5 (13.5)
		Bachelor’s degree	17 (43.6)	9 (23.7)
		Graduate’s degree	16 (41.0)	12 (31.6)
	**Marital status**			
		Single	2 (5.1)	3 (7.9)
		Partnered	1 (2.6)	2 (5.3)
		Married	33 (84.6)	28 (73.7)
		Divorced	3 (7.7)	3 (7.9)
		Widowed	0 (0.0)	2 (5.3)
	**Income (US $)^a^**			
		<20,000	3 (7.7)	5 (13.5)
		21,000-40,000	3 (7.7)	5 (13.5)
		41,000-60,000	5 (12.8)	7 (18.9)
		>61,000	28 (71.8)	20 (54.1)
**Chronic conditions**				
		Anxiety	10 (25.6)	12 (31.6)
		Hypertension	8 (20.5)	8 (21.1)
		Arthritis/rheumatic disease	7 (17.9)	5 (13.2)
		Depression	6 (15.4)	8 (21.1)
		Asthma	3 (7.7)	3 (7.9)
		Hypercholesterolemia	3 (7.7)	4 (10.5)
		PTSD^c^	2 (5.1)	1 (2.6)
		Diabetes	1 (2.6)	3 (7.9)
		Heart disease	1 (2.6)	0 (0.0)
		Other	9 (23.1)	4 (10.5)
		None	1 (2.6)	8 (21.1)

^a^Due to nonresponse, n=37 for ethnicity and income among those who did not complete the study. Percentages reflect percent of valid responses.

^b^GED: General Educational Development.

^c^PTSD: posttraumatic stress disorder.

### Outcomes

#### Use

**Figure 4:**
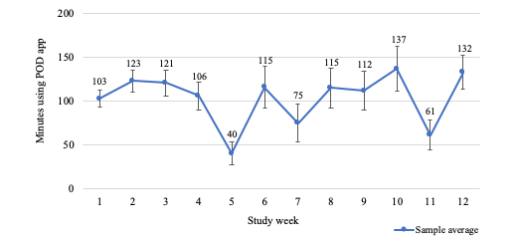
Average minutes using podcast app by week.

**Figure 5:**
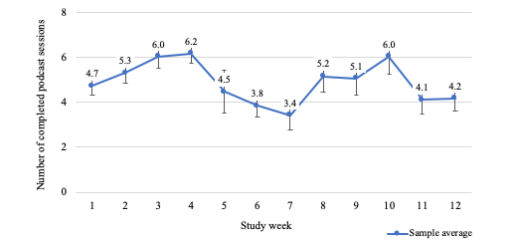
Average podcast sessions completed by week.

**Figure 6:**
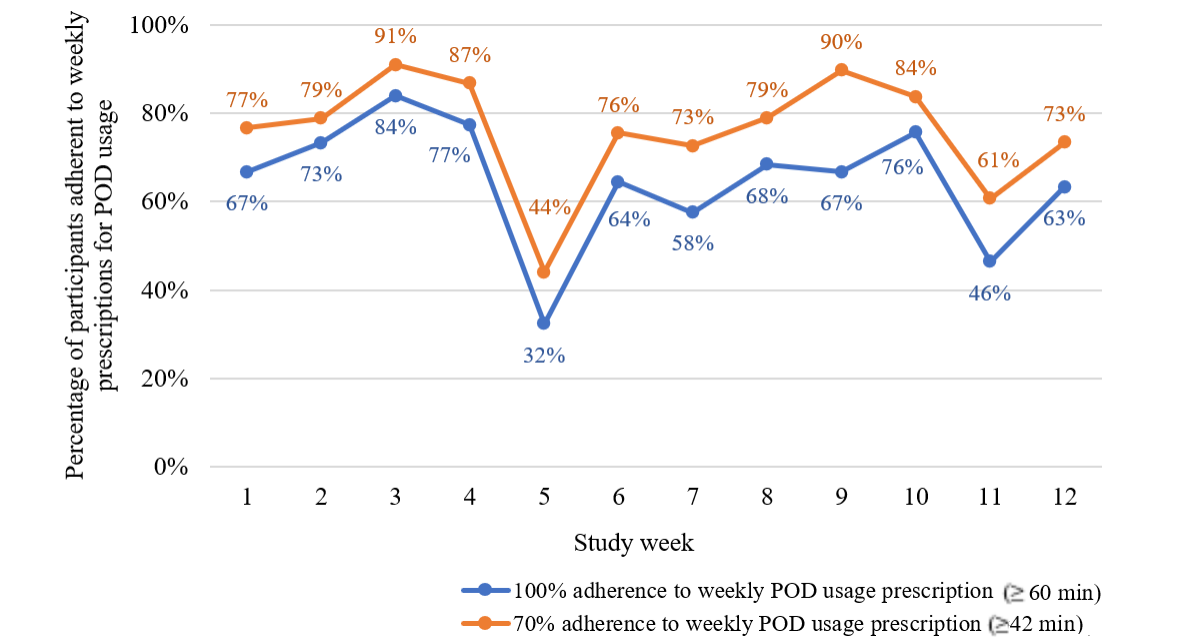
Percent adherence to prescribed podcast app use by week.

On average, participants listened to the health education podcasts for 103.2 (SD 29.5) minutes per week (see Figure 4), translating to an average of 4.9 (SD 0.9) completed podcast sessions each week (see Figure 5). For 83.3% (10/12) of the weeks, at least 70% of participants completed at least 70% of their total prescribed use (ie, 42 min/week; see Figure 6).

#### Satisfaction

Because we were unable to obtain satisfaction survey data from those who discontinued the study, satisfaction survey data were available only for study completers (n=39). Almost half of participants reported that, overall, they enjoyed the health education podcasts (n=19, 48.7%) and that they were satisfied with the intervention (n=17, 43.6%). Approximately half indicated that they learned something about their MPN or cancer in general from the podcasts (n=21, 53.8%), and 51.3% (n=20) reported that they made changes to their normal daily activities because of something that they learned in the podcasts. More than half of participants (n=22, 56.4%) indicated that they would recommend the 12-week podcast prescription to other patients with MPN.

Over half of the 39 participants (n=20, 51.3%) experienced some form of logistical limitation during the intervention. Specifically, 4 (10.3%) participants reported that they had difficulty hearing or viewing the podcasts (eg, too small on the screen), and 1 (2.6%) reported bad or slow internet connection. However, most frequently, participants indicated that they had “other” problems that were not available as survey response options (n=15, 38.5%). Of the 15 participants who provided open-ended responses describing their “other” difficulties, the most common were general problems with app functionality (n=7, 46.7%), problems accessing specific podcasts (n=5, 33.3%), or problems accessing any app content (ie, could not engage with any podcast; n=3, 20.0%). When asked if there was anything else they would like to share with us, the majority (21/34, 62%) that responded to this question made recommendations for changes to the podcast content, either to make it more specific to MPN or because they did not enjoy certain podcasts.

#### Changes in Health and Cancer-Related Symptoms

As shown in Table 2, using the health education podcasts was not associated with significant changes in global health, specific cancer-related symptoms, or MPN-SAF TSS.

**Table 2:** Changes in health and cancer-related symptoms during and after the health education podcast intervention (n=37).

Outcome		Baseline, mean (SD)	Week 6, mean (SD)	Week 12, mean (SD)	*F* test (*df*)^a^		*P* value^a^		Partial η^2^	
**Global health**						0.29 (1,36)		.88		0.033
	Physical health	33.24 (7.22)	33.08 (7.50)	33.62 (7.75)	0.46 (1,36)		.50		0.012	
	Mental health	40.01 (7.65)	39.76 (8.29)	39.63 (8.53)	0.27 (1,36)		.61		0.007	
**Cancer-related symptoms**						0.53 (1,36)		.83		0.127
	Anxiety	51.25 (8.00)	52.99 (7.51)	52.52 (7.29)	1.54 (1,36)		.22		0.041	
	Depression	49.46 (7.54)	48.81 (6.83)	49.71 (7.11)	0.04 (1,36)		.84		0.001	
	Pain intensity	41.77 (7.44)	41.56 (8.13)	42.26 (8.42)	0.23 (1,36)		.64		0.006	
	Sleep disturbance	53.2 (8.62)	53.43 (8.55)	53.05 (8.56)	0.02 (1,36)		.90		<0.001	
MPN-SAF TSS^b^		24.97 (15.44)	22.58 (15.63)	22.71 (16.00)	1.99 (1,36)		.17		0.051	

^a^*F* test and *P* values assume symptom change to be a linear trend over time. Examination of change as a quadratic function produced similar results. For 2 participants, complete data were unavailable; they were excluded from the multivariate analyses.

^b^MPN-SAF TSS: Myeloproliferative Neoplasm Symptom Assessment Form Total Symptom Score.

## Discussion

### Principal Results

The purpose of this study was to beta test a health education podcast control group delivered via a smartphone app to inform the development of a comparator that could be used in future app-based meditation intervention studies. Feasibility benchmarks were met for demand (ie, for 10 of the 12 weeks at least 70% of participants listened to at least 42 min/week of podcasts); although, the attrition should be noted as an important qualifier when considering engagement more broadly. Acceptability benchmarks were not met, as less than half of participants enjoyed the podcasts and were satisfied with the intervention. As expected, there were no changes in global health, cancer-related symptoms, or total symptom burden (ie, MPN-SAF TSS) over the 12 weeks.

### Comparison With Prior Work

Use benchmarks were set at ≥70% of participants listening to ≥70% (ie, 42 min/week) of the prescribed total weekly podcasts. In nearly all weeks (ie, 10 out of the 12 weeks) this benchmark was met. This is encouraging as a control group in which participants adhere to the study prescriptions allows researchers to control for nonintervention treatment effects such as time and attention [4]. Furthermore, participants averaged 103 min/week of podcast listening or viewing. Other studies that have tested mobile-app control groups have not reported weekly participation data [13,26]. In our research studies in which we used a commercially available meditation app in college students for 8 weeks [15] and to patients with MPN cancer for 4 weeks [16], average weekly participation in meditation on the app was ~38 min/week and ~71 min/week, respectively. Our findings are promising because the use of the podcast control app was comparable to the use levels in our intervention studies, supporting its feasibility for a control group with the same time and attention as a consumer-based mobile app meditation intervention.

Satisfaction benchmarks were not met, indicating that participants’ overall satisfaction with the app was lacking. Less than half of participants indicated that they enjoyed the health education podcasts and less than half indicated that they were satisfied with the intervention. This is likely due to the podcast topics and technical issues and difficulties that came up during the intervention related to the functionality and usability of the app. The majority of participants that responded to an open-ended question to provide additional feedback about the study reported that they thought the app content should be modified to include more MPN-specific education or higher quality podcasts. The podcasts selected for the control app were intended to provide more generic cancer-related health education and not MPN-specific education. A better understanding of the potential users’ content preferences with a more user-centered approach is necessary for future iterations of the control app, especially because the app was designed to be able to change health-education podcasts per target population being studied. A user-centered approach when developing products or software may help the user feel more at ease and make engaging with the content more intuitive, potentially improving adherence and enjoyment [27].

Over half of participants reported experiencing trouble accessing podcasts or content, or general problems with the functionality of the app. The app was not developed to be of commercial quality but was rather developed to be a “shell” of a design to be improved upon in subsequent developments after beta testing. Therefore, it is not surprising that such technical difficulties related to app functionality and accessibility were experienced. Specifically, there was a decrease in participation during week five, coinciding with participants reporting technical difficulties to the research staff. It is well known that user experience is a critical component to the success of mHealth apps and that apps must appeal to the motivations of the user [28]. For example, the unified theory of acceptance and use of technology (UTAUT) suggests that users’ expectations of how an app will perform and how much effort it takes to use the app will influence their intentions and behaviors [29-31]. Future iterations of this app will use the current findings and a model-driven approach (eg, the UTAUT) to inform the development of the next version of the app to be used as a comparator in a randomized control trial.

Importantly, there were no significant changes in health or cancer-related symptoms from baseline to postintervention. This is despite more than half of the participants reporting that they made changes to their normal daily activities because of something they learned from the podcasts. This is encouraging and indicates that this control group design is an appropriate time- and attention-matched condition that is not associated with any meaningful change in study outcomes. Education-based control groups have been used successfully as comparators in a range of smartphone-based interventions across different populations, typically without having significant effects on psychological or physical outcomes [13,16,26]. This indicates that an education-based control group could be appropriate for use in efficacy trials without producing changes in primary outcomes that are physical or psychological in nature. Future iterations of the app will be evaluated as a comparator in studies with other populations.

### Limitations

This study is not without its limitations. First, attrition during the study was high, such that only 51% (41/80) of participants completed the full 12-week intervention. Given that the podcast app use (minutes listened per week) was high even when including use of participants who did not complete the study, future research should collect more nuanced data on participant satisfaction to improve podcast app user experiences and reduce attrition rates. For example, the lack of a postintervention interview did not allow for deeper qualitative analyses into what participants liked and did not like related to the app. This qualitative data could have been a useful addition for gathering deeper insights into user satisfaction and informing future development of the app. Second, the sample was predominantly White, non-Hispanic, and female. This is not representative of the general population of patients with MPN and, more importantly for the purpose of this beta test, does not capture feedback that reflects the experiences of different genders, races, and ethnicities. Future feasibility research should aim to include more diverse samples to gather more representative feedback. Third, there was no comparative group, and this must be considered when analyzing the results. Fourth, offering a US $25 digital gift card for completion of all questionnaires could contribute to higher engagement rates. Researchers may need to account for an impact on their participant engagement if they do not provide similar incentivization. Finally, the use of cancer-specific educational content would limit the app as currently developed to use in cancer studies only. However, the content of the app can easily be adjusted to fit the needs of different populations while maintaining the integrity and functionality of the app to match the Calm meditation app.

### Future Research

Although this app was originally designed to be a comparative control app for app-based mindfulness meditation interventions involving Calm, the app is not reliant on the use of Calm and may be used as a control app in future app-based interventions. Currently, this app is not available to other researchers because it was only developed as a template to then create an improved comparator app for future mindfulness meditation app-based interventions based on the data collected. Once the app can be determined feasible, future interventions will be developed to assess app-based mindfulness meditation interventions as compared to the control app to assess many aspects of health and well-being across various populations.

### Conclusion

In summary, a 12-week mobile app health education podcast met the demand benchmark but not the acceptability benchmark for feasibility in a sample of patients with hematological cancer. Using the mobile app health education podcast was not associated with significant changes in cancer-related symptoms. Participants reported dissatisfaction with content and technical or functionality difficulties, which will be addressed in the future development of the health education podcast app. Based on findings from this study, a mobile app health education podcast may be a feasible option as a time- and attention-matched comparator condition for efficacy trials with more extensive formative research for the content of the podcasts and its acceptability by the specific population.

## Abbreviations

mHealth: mobile health
MPN: myeloproliferative neoplasm
MPN-SAF TSS: Myeloproliferative Neoplasm Symptom Assessment Form Total Symptom Score
NIH: National Institutes of Health
PROMIS: Patient-Reported Outcomes Measurement Information System
UTAUT: unified theory of acceptance and use of technology
